# Identification of potential DNA methylation biomarkers related to diagnosis in patients with bladder cancer through integrated bioinformatic analysis

**DOI:** 10.1186/s12894-023-01307-5

**Published:** 2023-08-10

**Authors:** Hongxia Cheng, Yuhua Liu, Gang Chen

**Affiliations:** School of Biological and Pharmaceutical Engineering, Wuhan Huaxia Institute of Technology, Wuhan, 430223 Hubei China

**Keywords:** Bladder cancer, DNA methylation, Diagnostic biomarker, Bioinformatic analysis

## Abstract

**Background:**

Bladder cancer (BLCA) is one of the most common malignancies among tumors worldwide. There are no validated biomarkers to facilitate such treatment diagnosis. DNA methylation modification plays important roles in epigenetics. Identifying methylated differentially expressed genes is a common method for the discovery of biomarkers.

**Methods:**

Bladder cancer data were obtained from Gene Expression Omnibus (GEO), including the gene expression microarrays GSE37817( 18 patients and 3 normal ), GSE52519 (9 patients and 3 normal) and the gene methylation microarray GSE37816 (18 patients and 3 normal). Aberrantly expressed genes were obtained by GEO2R. Gene Ontology (GO) and Kyoto Encyclopedia of Genes and Genomes (KEGG) pathways were analyzed using the DAVID database and KOBAS. Protein-protein interactions (PPIs) and hub gene networks were constructed by STRING and Cytoscape software. The validation of the results which was confirmed through four online platforms, including Gene Expression Profiling Interactive Analysis (GEPIA), Gene Set Cancer Analysis (GSCA), cBioProtal and MEXPRESS.

**Results:**

In total, 253 and 298 upregulated genes and 674 and 454 downregulated genes were identified for GSE37817 and GSE52519, respectively. For the GSE37816 dataset, hypermethylated and hypomethylated genes involving 778 and 3420 genes, respectively, were observed. Seventeen hypermethylated and low expression genes were enriched in biological processes associated with different organ development and morphogenesis. For molecular function, these genes showed enrichment in extracellular matrix structural constituents. Pathway enrichment showed drug metabolic enzymes and several amino acids metabolism, PI3K-Akt, Hedgehog signaling pathway. The top 3 hub genes screened by Cytoscape software were EFEMP1, SPARCL1 and ABCA8. The research results were verified using the GEPIA, GSCA, cBioProtal and EXPRESS databases, and the hub hypermethylated low expression genes were validated.

**Conclusion:**

This study screened possible aberrantly methylated expression hub genes in BLCA by integrated bioinformatics analysis. The results may provide possible methylation-based biomarkers for the precise diagnosis and treatment of BLCA in the future.

## Introduction

Bladder cancer (BLCA) is the most lethal malignancy of the urinary tract and the most common nonskin, solid cancer. In 2020, GLOBOCAN estimated 573278 new cases and 212536 deaths, making BLCA the tenth most diagnosed cancer worldwide [1, 2]. According to reports, with variable risks of recurrence and progression, the mortality and morbidity of BLCA have gradually increased in recent years [3, 4]. Transurethral resection of bladder tumor (TURBt) was the gold standard for the initial diagnosis and treatment of non-muscle invasive bladder cancer (NMIBC) [5]. Due to the high recurrence rate of NMIBC, patients need to undergo disproportionately invasive and unpleasant cystoscopy 4 times each year [6]. Therefore, a simple and reliable biomarker is necessary for accurate diagnosis of BLCA.

As heritable gene expression alterations, one of the most widespread epigenetic alterations is DNA methylation, which can affect the function of tumor suppressor genes and change their expression [7–13]. Because DNA methylation is conventionally regarded as a silencing epigenetic marker, several methylation markers have been reported in the detection of BLCA and prediction of the risk of disease prognosis and progression in recent years [14–16]. Hence, further research on methylated differentially expressed genes (MeDEGs) using high-throughput data has great significance for discovering novel cancer biomarkers. With the development of bioinformatics, many excellent software and online tools have emerged. These bioinformatics tools provided rapid and convenient analysis methods for the large amount of data from diverse gene-sequencing platforms and accurately screened potential novel genes as biomarkers [17, 18].

The existing literatures on DNA methylation considered imperfect because the analytical and validated methods used in these studies lacked systematicity and integrity. In this study, the potential biomarker which had strength relation with BLCA were screened from different database used a series of advanced bioinformatics tools. In addition, the results were identified by several online platform to ensure the validation. The aim of these research was to identify the hub MeDEGs that were greatly associated with BLCA. We hope that this research will provide valuable biomarker candidate genes for BLCA diagnosis.

## Materials and methods

### Microarray data collection

After a systematic search of the GEO database, two gene expression profiling datasets (GSE37817 public on May 03, 2013; GSE52519, public on Nov 20, 2013) and one gene methylation profiling dataset (GSE37816, public on May 03, 2013 ) were selected and downloaded from the Gene Expression Omnibus (https://www.ncbi.nlm.nih.gov/geo/) of The National Center for Biotechnology Information (NCBI). GSE37817 and GSE52519 were based on GPL6102 (Illumina human-6 v2.0 expression bead chip). GSE37816 was based on GLP8490 (Illumina HumanMethylation27 Bead Chip (Human Methylation27_270596_v.1.2)). GSE37817 consisted of 18 patients and 3 normal controls. GSE52519 consisted of 9 patients and 3 normal controls. GSE37816 consisted of 18 cancer patients and 6 controls.

### Data processing

GEO2R was an interactive web tool composed with GEOquery and limma. GEOquery parsed GEO data into R data structures. Limma (Linear Models for Microarray Analysis) was a statistical test to identify differentially expressed. GEO2R allowed users to compare two or more groups of samples in a GEO series in order to identify heatmapgenes that were differentially expressed across experimental conditions [19]. GEO2R was adopted to identify the differentially expressed genes (DEGs) between bladder cancer and non-bladder cancer tissues from GSE37817 and GSE52519. P < 0.05 and $|\log _{} FC |> 1$ were used as the cut-off criteria to find DEGs. The MeDEGs were identified from GSE37816 if they met the cut-off criteria of P < 0.05. The heatmap of the top 100 DEGs and MeDEGs was drawn using the heatmap online tool [20]. The intersecting genes were chosen using the Venn diagram web tool [21]. 

### Function and pathway enrichment analysis

Gene Ontology (GO) enrichment analysis included molecular function (MF), cellular component (CC), and biological process (BP) using DAVID (v2022q4). KOBAS (version 3.0) was applied for KEGG pathway enrichment [22]. A *P*-value < 0.05 was used as the cut-off to analyze the GO and pathway enrichment.

### PPI network construction and hub gene identification

The protein-protein interaction (PPI) network of hypermethylated-downregulated genes was constructed using STRING software. An interaction score of 0.3 was regarded as the cut-off criterion. The degree values were calculated by the Cytoscape (v3.9.1) plugin cytoHubba, and the top 10 were considered hub genes [23].

### Validation of chosen hub genes

The Cancer Genome Atlas (TCGA) collected, analyzed over 11,000 cancer samples from patients across 33 cancer types. Genotype-Tissue Expression (GTEx) produced RNA-Seq data for over 8000 normal samples, albeit from unrelated donors to balance the tumor data. GEPIA (Gene Expression Profiling Interactive Analysis) was a newly developed interactive web server for analyzing the RNA sequencing date for tens of thousands of cancer and non-cancer samples from the TCGA and the GTEx projects. Comparing with others web tools, GEPIA may provide detailed of 9736 tumors and 8587 normal samples differential expression analyses, chromosomal distribution plots, similar gene detection, dimensionality reduction analysis or expression comparison among pathological stages [24]. Gene Set Cancer Analysis (GSCA) integrated over 10,000 multi-dimensional genomic data across 33 cancer types from TCGA and over 750 small molecule drugs. This integrated platform provided a series of services to perform gene set expression, mutation and methylation analyses [25].

To validate the results, the interactive web server GEPIA was employed to compare the expression level of each hub gene between BLCA samples and normal samples. Furthermore, the GSCA platform for genomic cancer analysis was used to compare the methylation status of hub genes between BLCA samples and normal tissue samples.

The cBioPortal was a resource for interactive exploration of multidimensional cancer genomics data sets. Multiple types of genomic alteration data could be simultaneously displayed by cBioPortal [26, 27]. MEXPRESS was a data visualization tool designed for the easy visualization of DNA expression, DNA methylation and clinical data, as well as the relationships between them. The feature of this web tool was allowed you to look at DNA methylation data in relation to its genomic location [28].

To investigate the correlation between the methylation and expression of MeDEGs, the cBioPortal platform for exploration, visualization and analysis of BLCA genome data was used. The data of 413 BLCA patients were enrolled from TCGA. Finally, for the integration and visualization of relationships between DNA methylation and gene expression levels of hub genes, MEXPRESS visualization tool was exploited.

## Results

### Identification of MeDEGs

**Fig. 1:**
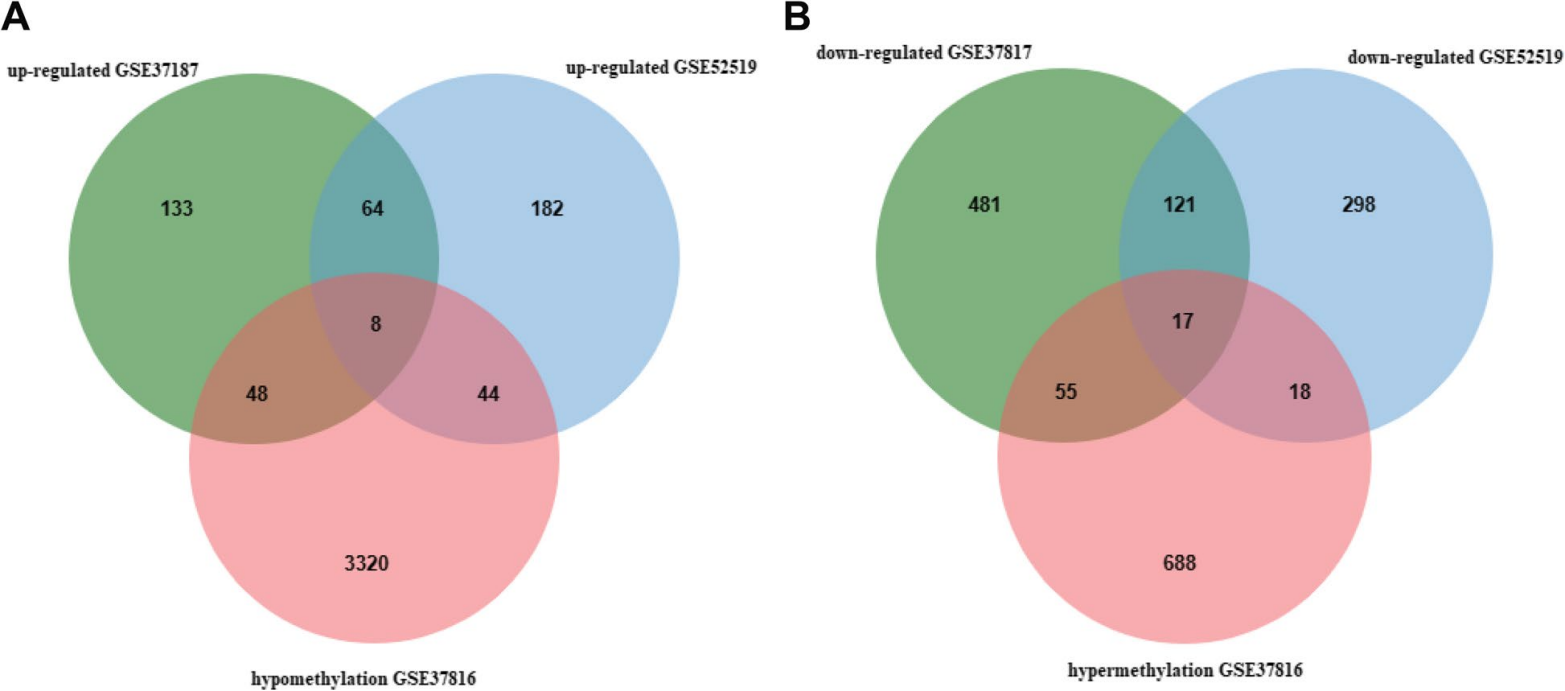
Identification of MeDEGs in gene expression profiling datasets (GSE37187, GSE52519) and gene methylation profiling datasets (GSE37186) (intersection of A: hypomethylated and highly regulated expression genes. Intersection of B: hypermethylated and downregulated genes)

**Fig. 2:**
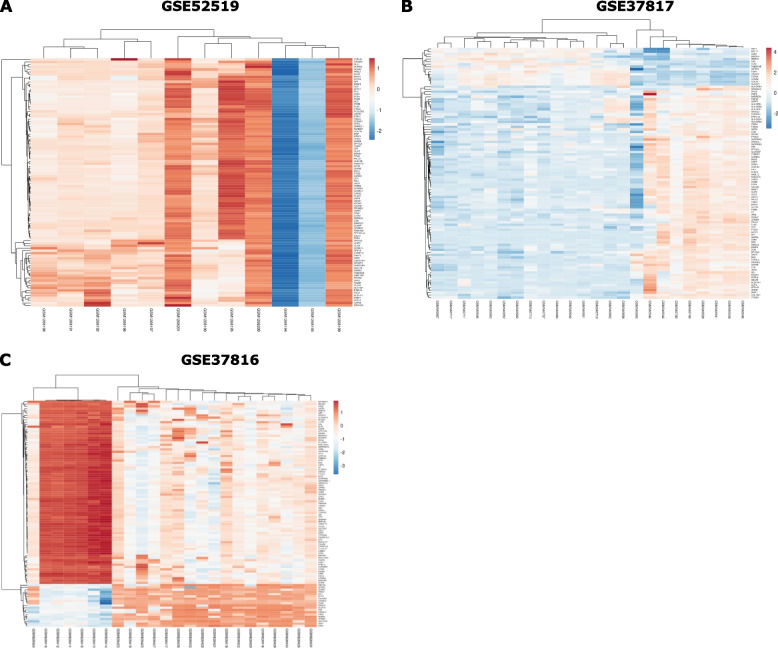
Heatmap of the top 100 DEGs (A, B) and MeDEGs (C)

In two gene expression profiling datasets, 72 genes were upregulated (298 in GSE52519 and 253 in GSE37817), and 138 genes were downregulated (674 in GSE37817 and 454 in GSE52519). In the gene methylation profiling dataset, there were 778 hypermethylated genes and 3420 hypomethylated genes. Using a Venn diagram, 17 hypermethylated, low-expressing genes and 8 hypomethylated, high-expressing genes were identified (Fig. 1A-B). The top 100 DEGs and MeDEGs with the highest differences are illustrated on the heatmap in Fig. 2A-C.

### GO functional enrichment analysis of MeDEGs

Gene ontology (GO) enrichment analysis of MeDEGs using DAVID is illustrated in Table 1. For hypermethylated and downregulated genes, biological processes (BP) were mainly associated with different organ development and morphogenesis. For molecular function (MF), the results were enriched in extracellular matrix structural constituents. The cell component (CC) analysis indicated enrichment of 10 extracellular regions and the extracellular matrix.

**Table 1:** Gene ontology analysis of MeDEGs

Category	Term	Gene count	%	*P* value
Hypermethylation and low expression				
	GO:0009887 animal organ morphogenesis	6	35.29	1.11E-03
	GO:0048857 neural nucleus development	3	17.65	1.29E-03
	GO:0048732 gland development	4	23.53	5.63 E-03
	GO:0048592 eye morphogenesis	3	17.65	7.03E-03
	GO:0009790 embryo development	5	29.41	9.62 E-03
	GO:0048598 embryonic morphogenesis	4	23.53	0.012
	GO:0048729 tissue morphogenesis	4	23.53	0.013
	GO:0010224 response to UV-B	2	11.76	0.013
GOTERM_BP_FAT	GO:0090596 sensory organ morphogenesis	3	17.65	0.020
	GO:0048562 embryonic organ morphogenesis	3	17.65	0.024
	GO:0045596 negative regulation of cell differentiation	4	23.53	0.025
	GO:0007420 brain development	4	23.53	0.026
	GO:0048048 embryonic eye morphogenesis	2	11.76	0.028
	GO:0060322 head development	4	23.53	0.030
	GO:0001654 eye development	3	17.65	0.039
	GO:0033993 response to lipid	4	23.53	0.042
	GO:0031103 axon regeneration	4	23.53	0.042
GOTERM_MF_FAT	GO:0005201 extracellular matrix structural constituent	3	17.65	9.69 E-03
GOTERM_CC_FAT	GO:0031012 extracellular matrix	3	17.65	0.039
	GO:0005576 extracellular region	9	52.94	0.043
	GO:0044421 extracellular region part	8	47.06	0.047

**Fig. 3:**
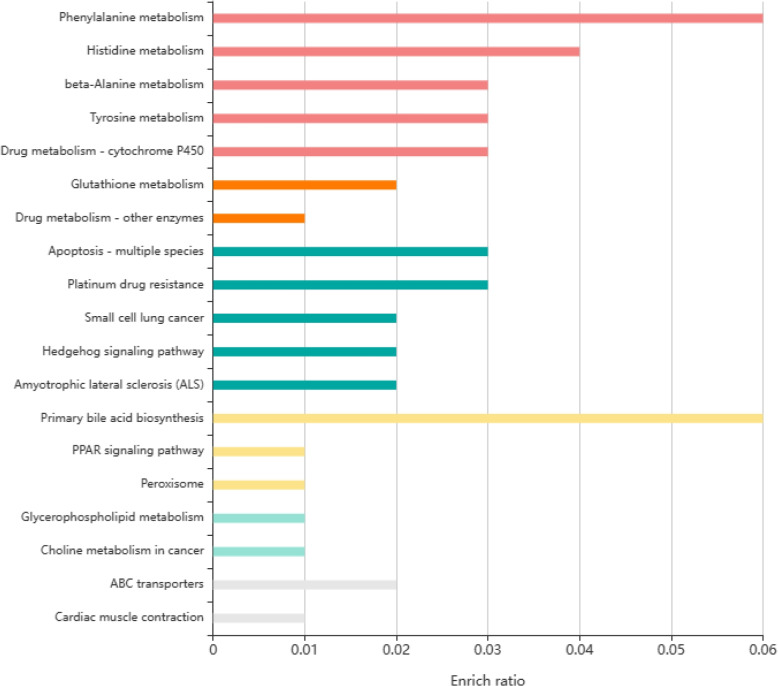
KEGG pathway enrichment of hypermethylated genes in bladder cancer

KEGG pathway enrichment analysis indicated that hypermethylated and low expression genes were significantly enriched in metabolism related to the drug metabolic enzyme CYP450 and several amino acids, signaling pathways related to PPAR (peroxisome proliferator-activated receptor, PPARs), PI3K-Akt, and Hedgehog. Enriched terms visualized in barplot using KOBAS. The results are shown in Fig. 3 and Table 2.

**Table 2:** KEGG enrichment of hypermethylated and low expression genes ($P<$0.01)

Term	ID	*P*-Value
Metabolic pathways	hsa01100	2.80e-4
Drug metabolism - cytochrome P450	hsa00982	4.68e-4
Platinum drug resistance	hsa01524	4.81e-4
Metabolism of xenobiotics by cytochrome P450	hsa00980	5.20e-4
Chemical carcinogenesis	hsa05204	6.02e-4
Small cell lung cancer	hsa05222	7.70e-4
Toxoplasmosis	hsa05145	1.12e-3
Pathways in cancer	hsa05200	1.47e-3
Fluid shear stress and atherosclerosis	hsa05418	1.68e-3
Focal adhesion	hsa04510	3.37e-3
Phenylalanine metabolism	hsa00360	7.77e-3
Primary bile acid biosynthesis	hsa00120	7.77e-3
PI3K-Akt signaling pathway	hsa04151	1.00e-2

### PPI network construction and hub gene selection

**Fig. 4:**
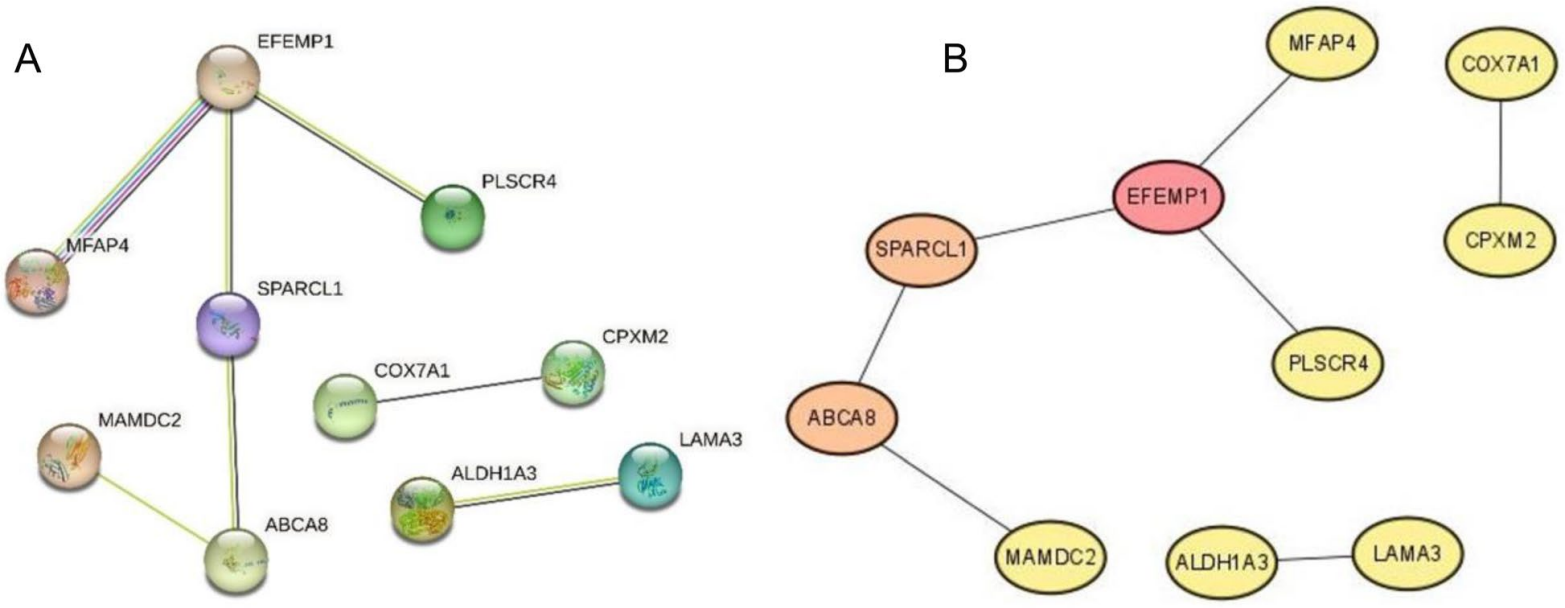
PPI and hub gene identification of hypermethylated-downregulated genes (A: PPI. B: The hub genes were screened by Cytoscape)

Protein-protein interaction (PPI) networks were constructed using the STRING database. The PPI network for hypermethylated and low expression genes is shown in Fig. 4A. The degree of all nodes was calculated by the Cytoscape plugin cytoHubba. Genes with higher degree values were considered hub genes. The order of hub genes was EFEMP1, SPARCL1, ABCA8, ALDH1A3, CPXM2, COX7A1, MAMDC2, MFAP4, PLSCR4 and LAMA3. The network of hub genes is illustrated in Fig. 4B.

### Validation of the top 10 hypermethylated low expression genes

**Fig. 5:**
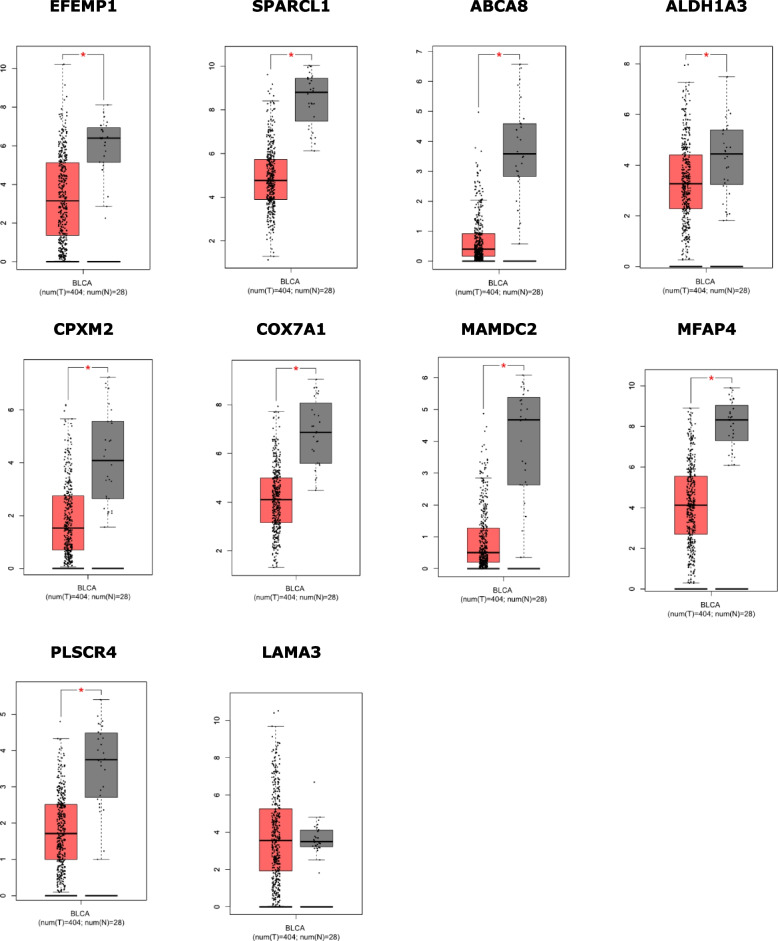
Comparison of the hub gene expression in BLCA and normal tissues using GEPIA. (Tumor samples are marked in red, and normal samples are marked in gray. *P < 0.01 was considered statistically significant)

First, the expression statuses of 10 hub genes were compared between normal and BLCA tissues in TCGA and GTEx database using the GEPIA online platform. *P*-value cutoff was 0.01. The results are shown in Fig. 5. From the results, except for LAMA3, the other gene expression levels in BLCA were significantly lower than those in normal tissue.

In addition, the multiple gene expression comparison was also executed. The results shown that the SPARCL1, MFAP4, COX7A1, EFEMP1 and MAMDC2 were highly expressed in normal tissue among the hub genes. By compared, MFAP4, MAMDC2, SPARCL1, ABCA8 and COX7A1 had significant differential expression between tumors or normal tissues in BLCA.

**Fig. 6:**
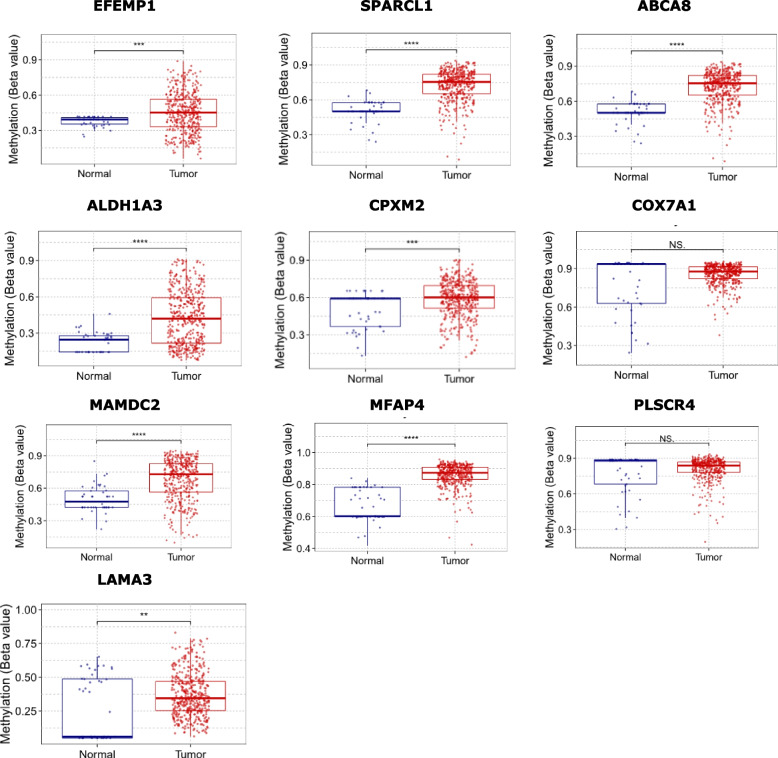
Comparison of the methylated expression statuses of hub genes between BLCA tissue and normal tissues using GSCA. (Tumor samples are marked in red, and normal samples are marked in blue. *FDR<0.01 was considered statistically significant)

Furthermore, the methylated expression statuses of hub genes were compared between normal and BLCA tissues using GSCA online platform. The *p*-value was estimated by t-test and was further adjusted by FDR. The cutoff was FDR $\le$ 0.05. The outcome is summarized in Fig. 6. From the figure, except for LAMA3, the methylated expression level in tumor tissues was significantly higher than that in normal tissue. The highly methylated level between tumor and normal tissues were ALDH1A3, EFEMP1, SPARCL1, CPXM2 and EFEMP1.

**Fig. 7:**
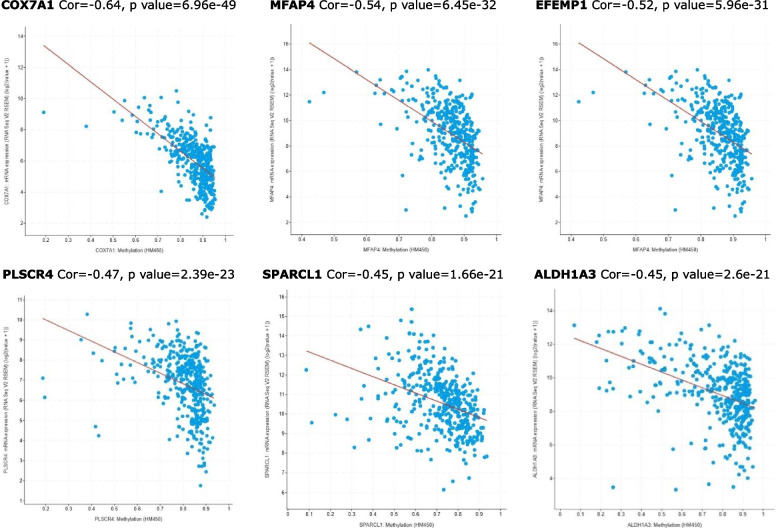
Spearman’s correlation analysis between gene expression level and methylated expression level of COX7A1, MFAP4, EFEMP1, PLSCR4, SPARCL1 and ALDH1A3 genes. (Spearman’s correlation coefficient and *P*-values are shown in each plot)

**Fig. 8:**
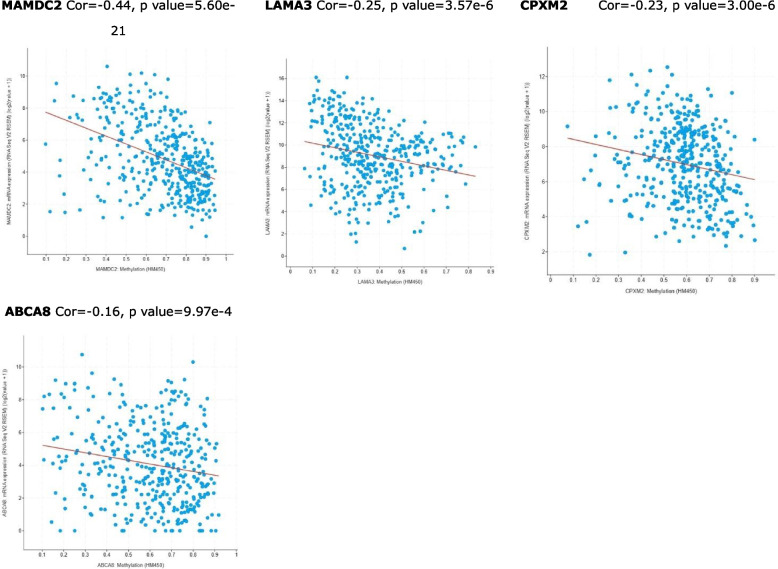
Spearman’s correlation analysis between gene expression level and methylated expression level of MAMDC2, LAMA3, CPXM2, ABCA8 genes. (Spearman’s correlation coefficient and *P*-values are shown in each plot)

The correlation between the mRNA expression levels and methylation expression was performed using the cBioPortal online platform. Spearman’s analysis results are illustrated in Figs. 7-8. Obviously, the correlation between the mRNA expression and methylated expression was negative among the hub genes. The co-efficient was medium level in COX7A1, EFEMP1 and MFAP4 (Cor>0.5).

**Fig. 9:**
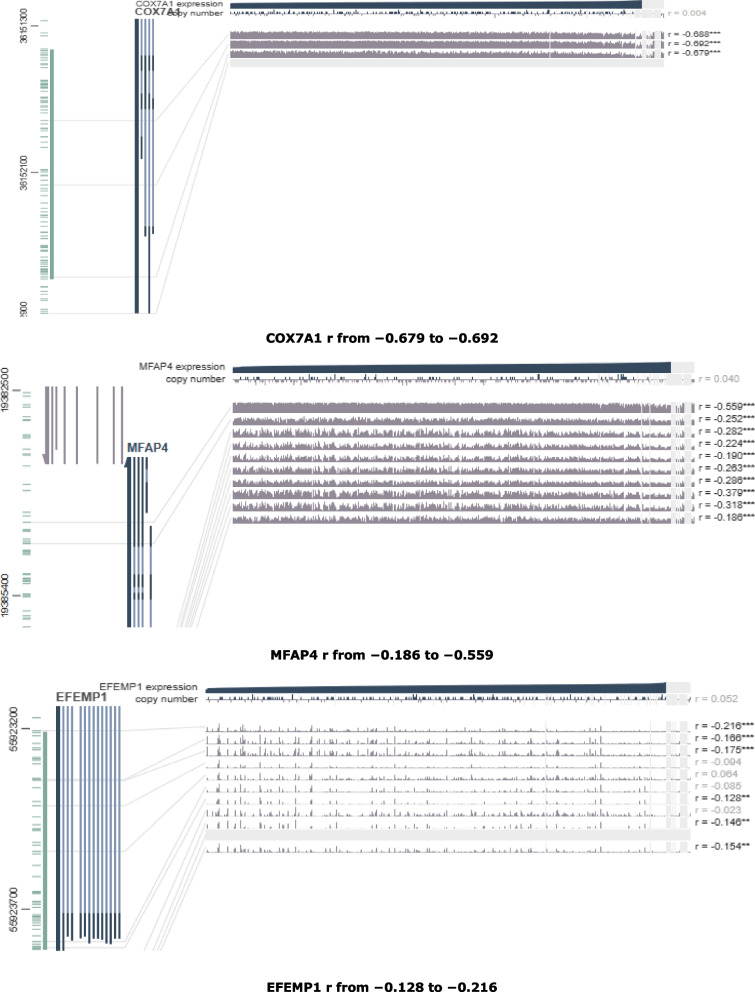
Visualization of the DNA of CpGs island methylated expression of COX7A1, MFAP4 and EFEMP1 genes in BLCA (Pearson correlation range around the promoter region were shown in each plot. *p < 0.05, **p < 0.01, ***p < 0.001)

**Fig. 10:**
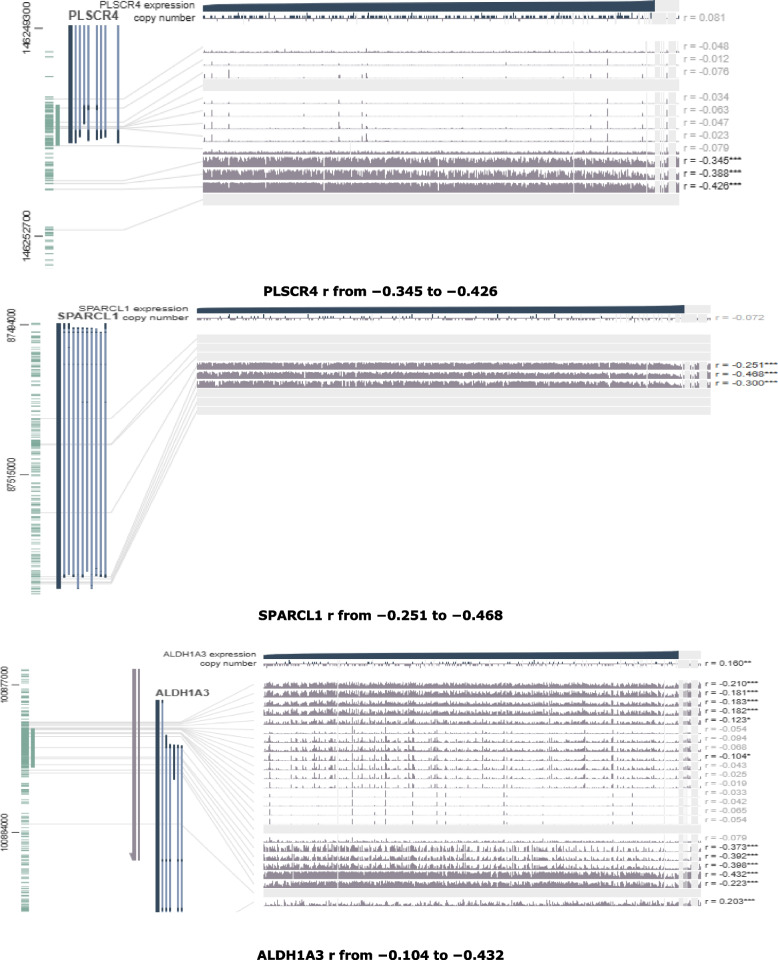
Visualization of the DNA of CpGs island methylated expression of PLSCR4, SPARCL1 and ALDH1A3 genes in BLCA (Pearson correlation range around the promoter region were shown in each plot. *p < 0.05, **p < 0.01, ***p < 0.001)

**Fig. 11:**
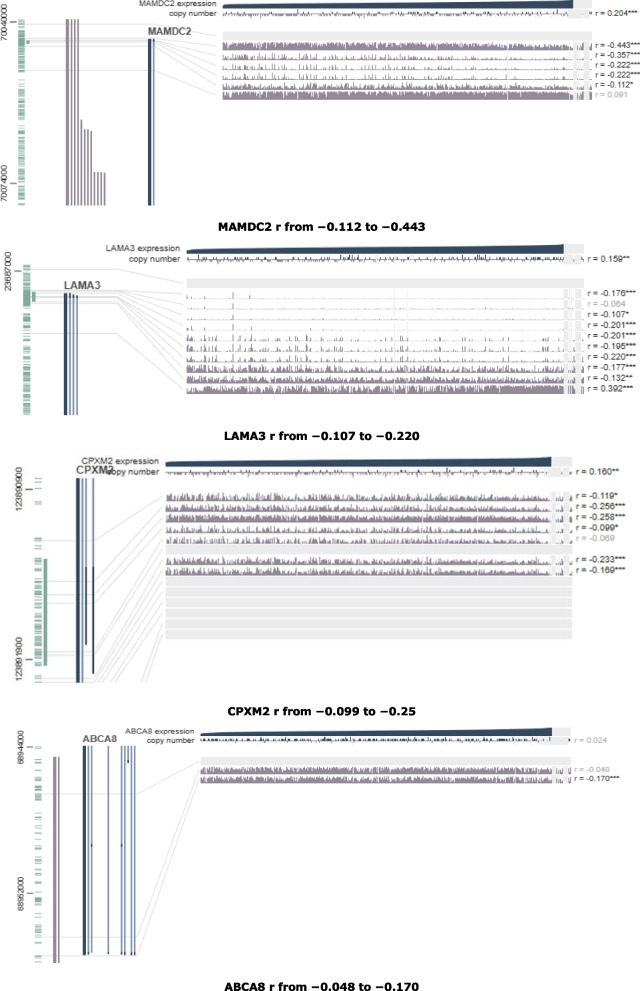
Visualization of the DNA of CpGs island methylated expression of MAMDC2, LAMA3, CPXM2 and ABCA8 genes in BLCA (Pearson correlation range around the promoter region were shown in each plot. *p < 0.05, **p < 0.01, ***p < 0.001)

Finally, the MEXPRESS tool was used to investigate the DNA methylation changes at individual CpGs in BLCA. From Figs. 9, 10 and 11, it was clear that the normal samples clustered towards higher expression. There was a negative correlation between expression and methylation around the promoter region.

## Discussion

BLCA is a common malignancy of the urinary tract and a significant cause of cancer morbidity and mortality worldwide. The five-year survival rate is only 5% in patients with distant metastasis [29].In recent years, some methods for predication postoperative survival and recurrent of BLCA were reported [30]. Epigenetic mechanisms take part in an important role in the pathogenesis of BLCA. Identifying accurate biomarkers for primary BLCA is a key clinical need for BLCA diagnostics. At meantime, the effective biomarkers are also important for the therapy of BLCA and healthcare [31, 32]. Many studies have exploited aberrant DNA expression signatures or methylation signatures to predict the characteristics or prognosis and drug resistance of different type cancer, such as BLCA [33–35] and prostate cancer [36, 37].

In this study, several bioinformatics analysis methods were applied to identify potential key MeDEGs associated with BLCA. Using two DEG profiles of BLCA obtained from the GEO database, 72 upregulated and 138 downregulated DEGs were observed. By comparing the MeDEG profile retrieved from the GEO database with these DEGs, 8 hypomethylated and highly expressed genes and 17 hypermethylated and lowly expressed genes were identified.

GO enrichment analysis showed that hypermethylated and low expression genes were mainly enriched in organ development and morphogenesis-related BP, especially in neural nucleus and gland development. KEGG enrichment analysis indicated that metabolism for CYP450, several amino acids metabolism and signaling pathways were significantly enriched. Interestingly, these signaling pathways and substances which were closely related to cell proliferation and the pharmacodynamics of antitumor drugs. For instance, PI3K-Akt activation was also found in breast cancer [38], gastric cancer [39], and thyroid carcinoma [40]. Activation of Hedgehog (Hh) signal resulted in tumorigenesis, malignancy, such as basal cell carcinoma, pancreatic cancer, prostate cancer [41–43]. Hypermethylated genes were also related to focal adhesion in the research, which potentially promotes tumor cell proliferation and mobility [44].

The 17 hypermethylated low expression genes, including ISL1, ABCA8, MFAP4, COX7A1, SPARC1, ALDH1A3, ACOX2, HOXA9, PLSCR4, CPXM2, BCL2, MAMD2, CKB, EFEMP1, SNRPN, GSTM5, and LAMA3 were analyzed using Cytoscape software. EFEMP1, SPARCL1, ABCA8, MFAP4, PLSCR4, MAMDC2, COX7A1, CPXM2, ALDH1A3 and LAMA3 were identified as hub genes. Among these genes, ALDH1A3, HOXA9 and ISL1 methylation patterns have been reported to be related to the clinical outcomes of BLCA [45–47]. SPARCL1 was a prognostic biomarker for colorectal cancer because its expression was downregulated through DNA methylation [48, 49]. Many genes such as ABCA8, MFAP4 and MAMDC2 also been potential diagnostic and prognostic biomarkers in hepatocellular carcinoma, breast cancer and ovarian cancer [50–53]. Because these genes were related to BLCA at the mechanistic level, it was possible to be a potential biomarker for BLCA.

The most of chosen hub genes were correct by four online platform tools validated. Through multiple genes comparison using the GEPIA online platform, the MFAP4, MAMDC2, SPARCL1, ABCA8 and EFEMP1 had highly difference expression level between tumors and normal tissue. Among these five genes, the SPARCL1, EFEMP1 and MFAP4 had significant highly methylation between normal and tumor tissues using GSCA online platform. The co-efficient >-0.5 between the mRNA expression levels and methylation expression were COX7A1, EFEMP1 and MFAP4. Through analysis, the MFAP4, SPARCL1, EFEMP1, COX7A1, ABCA8 and MAMDC2 would be more likely to become potential biomarker.

As was well known, CpGs were hot-shot regions of the genome, one-third of all point mutations causing genetic diseases in human result from mutation at CpG site [54]. The DNA methylation was changed during the initiation and progression of cancer with hypomethylation of CpG poor intergenic regions and hypermethylation of CpG islands associated with gene silencing and reduced plasticity [55]. In the genome of normal cells, promoter CpG islands were hypomethylated. However, tumor cell hypermethylation of the CpG island in the tumor suppressor promoter region was associated with malignant formation and progression [56, 57]. The methylation alternation of hub genes in BLCA and normal tissues were compared using MEXPRESS visualization tool. The results illustrated there were significant negative correlation in expression and methylation around the CpG and promoter region. The hypermethylation around promoter and CpG region of hub genes may led to down-regulate expression. The hub genes were related with PI3K-Akt and Hedgehog signal transduction which were also associated with cancer cell proliferation and survival. Hence hypermethylation would be associated with hub gene repression and initiate BLCA.

## Conclusion

In this study, several differentially methylated genes associated with BLCA were identified. The characteristics of the signatures were confirmed by a series of systematic bioinformatics analysis tools. We hoped these genes, especially the MFAP4, SPARCL1, EFEMP1, COX7A1, ABCA8 and MAMDC2, would be an effective biomarker for BLCA diagnostics.

This study was mainly based on bioinformatic analysis of the GEO database. The amount of data and verification of identified genes were insufficient. In addition, some of hypermethylated genes had been observed not only in BLCA but also in many other cancers. Future research will be needed to confirm the performance of these aberrantly methylated genes in clinical practice.

## Data Availability

The datasets generated during the current study are available in the GEO database (https://www.ncbi.nlm.nih.gov/geo/).
